# Pre-arterialization of coronary veins prior to retroperfusion of ischemic myocardium: percutaneous closure device

**DOI:** 10.3389/fcvm.2023.1208903

**Published:** 2023-09-18

**Authors:** Jenny S. Choy, Terry Hubbard, Eugene M. Golts, Deepak L. Bhatt, José A. Navia, Ghassan S. Kassab

**Affiliations:** ^1^ Department of Biomedical Engineering, California Medical Innovations Institute, San Diego, CA, United States; ^2^ 3DT Holdings, LLC, San Diego, CA, United States; ^3^ Division of Cardiovascular and Thoracic Surgery, University of California, San Diego, CA, United States; ^4^ Icahn School of Medicine at Mount Sinai Health System, New York, NY, United States; ^5^ Department of Cardiac Surgery, Austral University, Pilar, Buenos Aires, Argentina

**Keywords:** myocardial ischemia, coronary venous retroperfusion, venous remodeling, venous closure device, venous arterialization

## Abstract

**Background:**

Chronic coronary retroperfusion to treat myocardial ischemia has previously failed due to edema and hemorrhage of coronary veins suddenly exposed to arterial pressures. The objective of this study was to selectively adapt the coronary veins to become arterialized prior to coronary venous retroperfusion to avoid vascular edema and hemorrhage.

**Methods and results:**

In 32 animals (Group I = 19 and Group II = 13), the left anterior descending (LAD) artery was occluded using an ameroid occlusion model. In Group I, the great cardiac vein was blocked with suture ligation (Group IA = 11) or with occlusion device (Group IB = 8) to arterialize the venous system within 2 weeks at intermediate pressure (between arterial and venous levels) before a coronary venous bypass graft (CVBG) was implemented through a left internal mammary artery (LIMA) anastomosis. Group II only received the LAD artery occlusion and served as control. Serial echocardiograms showed recovery of left ventricular (LV) function with this adaptation-arterialization approach, with an increase in ejection fraction (EF) in Group I from 38% ± 5% after coronary occlusion to 53% ± 7% eight weeks after CVBG, whereas in Group II the EF never recovered (41% ± 2%–33% ± 7%). The remodeling of the venous system not only allowed restoration of myocardial function when CVBG was implemented but possibly promoted a novel form of “collateralization” between the native arterioles and the newly arterialized venules, which revascularized the ischemic myocardium.

**Conclusions:**

These findings form a potential rationale for a venous arterialization-revascularization treatment for the refractory angina and the “no-option” patients using a hybrid percutaneous (closure device for arterialization)/surgical approach (CVBG) to revascularize the myocardium.

## Introduction

Heart disease continues to be the leading cause of death in the US and worldwide (1), with coronary artery disease (CAD) being the most common type of heart disease. Currently, percutaneous coronary intervention (PCI) and coronary artery bypass grafting (CABG) are the standard methods for revascularization of the myocardium (2). Patients with diffuse CAD and a history of repeated coronary stents, however, are poor candidates for CABG or PCI. These “no-option” patients account for 14%–16% of the population requiring but ineligible (3, 4) for traditional myocardial revascularization therapy (2, 5). These patients with advanced CAD typically suffer from refractory angina and are in need of new therapeutic options.

Cardiovascular surgeons have long recognized the ideal nature and potential of coronary veins, which do not develop atherosclerosis (6), for alternative revascularization of the ischemic myocardium. The main limitation, however, has been the edema and hemorrhage of veins and post-capillary venules when suddenly exposed to arterial pressure (7, 8).

Sinus pressure elevation for the treatment of myocardial ischemia and the relief of angina has been extensively evaluated (9, 10). More recently, the Reducer (11), a device that narrows the coronary sinus and elevates pressure in the coronary venous system, was introduced (12) for the treatment of angina pectoris in patients who are not candidates for revascularization.

Our group has developed an experimental strategy to selectively arterialize the coronary venous system by ligation of the great cardiac vein (GCV) to increase venous pressure (13, 14) prior to retroperfusion for revascularization of the ischemic myocardium. This remodeling procedure (venous wall thickening) prepares the veins at an intermediate pressure (40–50 mmHg, between arterial and venous levels) for a two-week period prior to exposure to full arterial pressure. The approach avoids abruptly raising the pressure in the veins from venous (10–20 mmHg) to arterial values (100–120 mmHg) in a single step. Once the venous system is fully remodeled, resembling arterial vessels (arterialization), it can be used to retroperfuse the myocardium by anastomosing the arterial and venous systems and allowing oxygenated blood and nutrients to reach the ischemic territory in patients unsuitable for revascularization, using their own arterial pulse pressure.

The objective of this study was to present a novel proof of concept that coronary veins subjected to venous ligation (13, 14) and hence pressure elevation for two weeks, prior to retroperfusion, can become fully arterialized and serve to nourish the ischemic myocardium via coronary venous bypass graft (CVBG) in a chronic swine model. Furthermore, we hypothesize that retroperfusion of an arterialized venous system promotes a novel form of interconnections with the native arterial vessels to revascularize the ischemic myocardium. This novel form of “collateralization” dramatically augments the native collaterals to restore perfusion of the ischemic myocardium, and hence cardiac function.

To clinically translate these findings, we have developed and tested a venous pressure preconditioning (VPP) device that can be delivered percutaneously. The device is designed to create a focal plane of occlusion to minimize the degree of thrombosis in the venous system. This percutaneous device in conjunction with CVBG provides a clinical paradigm for treatment of no-option patients.

## Methods

### Animal preparation

All animal experiments were performed in accordance with the NIH and local ethical guidelines, including the Guide for the Care and Use of Laboratory Animals, the Public Health Service Policy on Humane Care and Use of Laboratory Animals, and the Animal Welfare Act, and an approved Indiana University Purdue University Indianapolis and California Medical Innovations Institute IACUC protocols regarding the use of animals in research.

**Figure 1:**
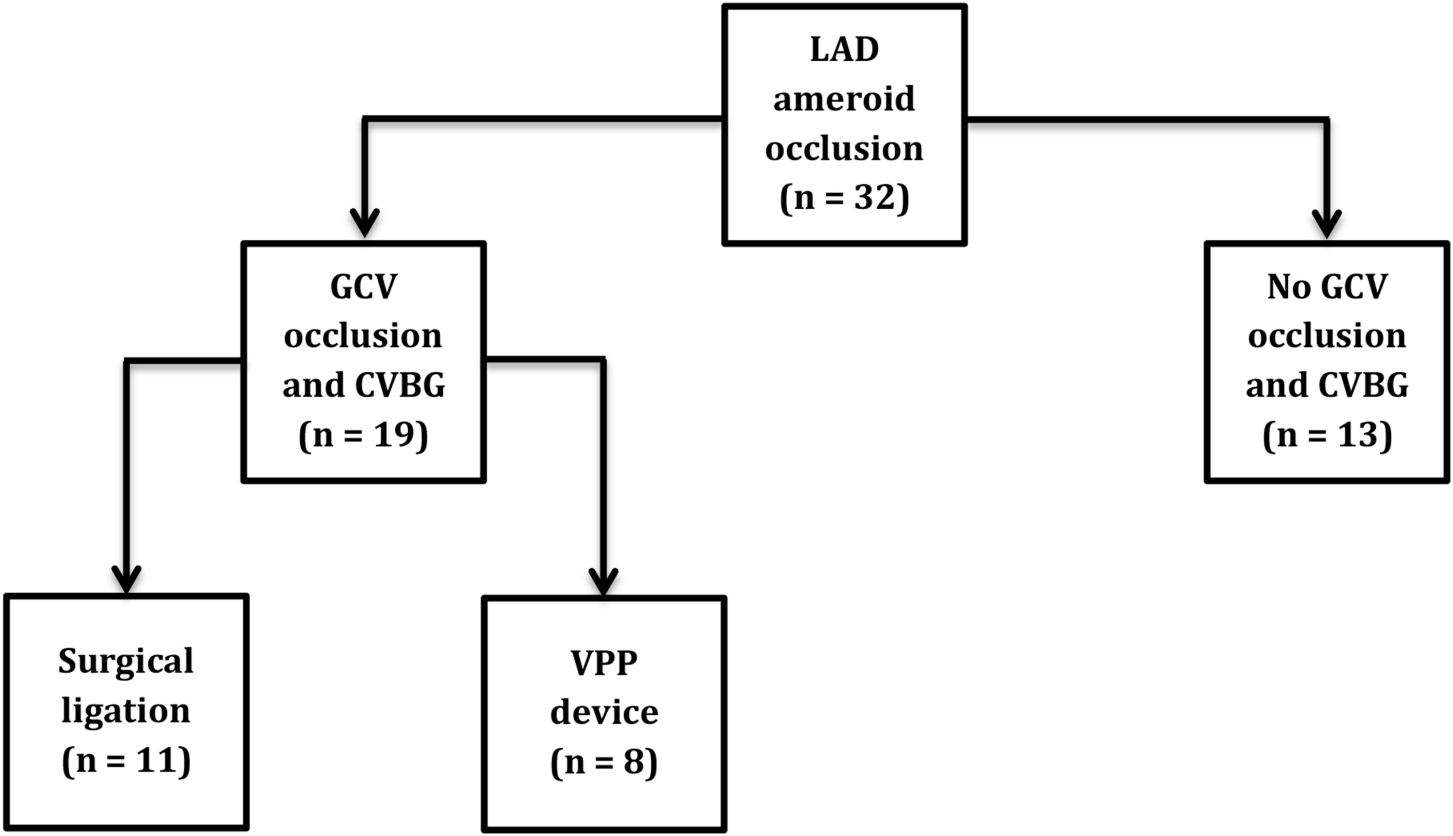
Schematic of the animal groups.

Thirty-two Yorkshire domestic swine (divided in 2 groups) of either sex, with body weight of 59.4 ± 8 kg were used in this study. Group IA (*n* = 11) had LAD artery occlusion, GCV surgical ligation, and CVBG. Group IB (*n* = 8) had LAD artery occlusion, GCV occlusion with VPP device, and CVBG. Group II (*n* = 13) only had LAD artery occlusion and served as control (Figure 1). The surgical ligation experiments were performed at Indiana University – School of Medicine Facilities while the VPP device experiments were performed at California Medical Innovations Institute. The pigs were fasted overnight. Sedation was achieved with TKX (Telazol 10 mg/kg, Ketamine 5 mg/kg, Xylazine 5 mg/kg; IM) and surgical anesthesia was maintained with isoflurane 1%–2% by inhalation through endotracheal tube. At the end of the study, the animals were euthanized under deep anesthesia (isoflurane 5%) with a bolus injection (60 ml) of a saturated solution of potassium chloride via the jugular vein to arrest the heart in diastole.

### LAD artery ameroid placement and GCV occlusion

The following procedures were performed in both groups (*n* = 32). Under sterile conditions, an introducer sheath was percutaneously inserted into the right jugular vein for administration of fluids and drugs. The chest was opened through a median-sternotomy and an incision of approximately 2 inches was made in the pericardium to access the LAD artery and the GCV. The LAD artery was dissected free from the surrounding tissue distal to the second diagonal branch and a 4-mm-diameter ameroid constrictor was placed around the artery. The ameroid constrictor consists of an inner ring of casein surrounded by a stainless steel sheath. Casein absorbs body fluid and swells inwardly over approximately 14 days, gradually closing the ring, obliterating the artery (nearly 100% stenosis), and creating an ischemic region in the LAD territory. The left atrium was then gently lifted with an atraumatic tissue clamp to expose the GCV. After either surgical suture ligation or percutaneous placement of VPP device (described below), the animals in Group I (treated group) were recovered for two weeks prior to a CVBG, while the animals in Group II (control group) survived for 10 weeks prior to terminal procedure.

### Vein suture ligation

In eleven animals (Group IA), the GCV was permanently ligated at the intersection between the anterior longitudinal sulcus and the base of the heart, with non-absorbable suture, to increase the venous pressure to approximately 50 mmHg in the territory that runs from apex to base, and to allow the venous system to remodel functionally (13, 14). Venous pressure elevation was confirmed before closing the chest, as previously described (13). Measurements were taken in the GCV at the anterior longitudinal sulcus, below the suture ligation.

### Percutaneous delivery of VPP device

**Figure 2:**
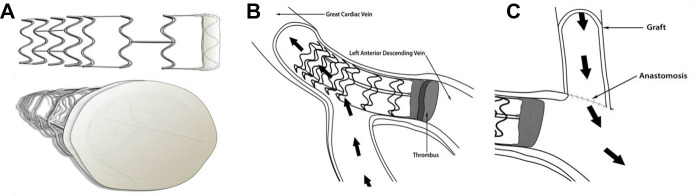
Frame design of the VPP device with open ring configuration (**A**) that prevents obstruction of side branches (**B**) to enable focal remodeling of the GCV in preparation for CVBG (**C**).

**Figure 3:**
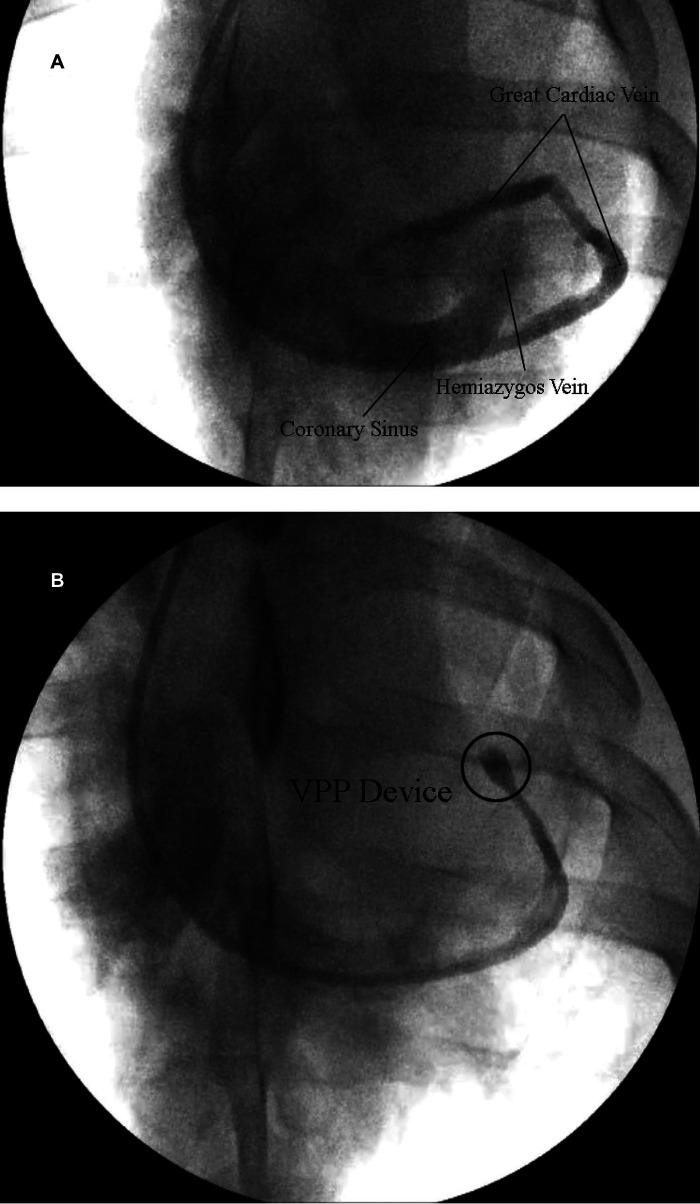
(**A**) Representative venogram of the coronary sinus, hemiazygos vein and great cardiac vein. (**B**) Representative venogram of the GCV showing complete occlusion of the vein (cease of contrast) in the interventricular sulcus with the VPP device.

The suture ligation results from the *in vivo* studies were used to evaluate the efficacy of a novel percutaneous VPP device (Figure 2). The VPP device was deployed in eight animals (Group IB). A 9F introducer sheath was percutaneously inserted into the right jugular vein and a 6F hockey stick catheter was advanced over a hydrophilic 0.035” guidewire into the coronary sinus and the GCV. The guidewire was further advanced towards the apical region and the hockey stick was exchanged with the VPP deployment catheter (a modified Zilver® Vena™ system, Cook Medical, Bloomington, IN). Once in place in the GCV (at the intersection between the anterior longitudinal sulcus and the base of the heart), the VPP device was deployed by pulling back the outer shaft of the catheter. Deployment and occlusion of the vein was confirmed with fluoroscopy, as shown in Figure 3B.

### CVBG surgery via LIMA

Two weeks after ameroid placement, the animals in Group I underwent CVBG. By this time, the ameroid constrictor had already created nearly 100% stenosis of the LAD artery (corroborated by angiography). The animals in Group II did not undergo CVBG and served as control. Two days before the CVBG surgery was performed, the animals received daily Aspirin 325 mg, PO, and Clopidogrel 75 mg, PO. On the day of the procedure, the right femoral artery was percutaneously accessed with a 6F introducer sheath and connected to a pressure transducer (TSD104A – Biopac Systems, Inc, Goleta, CA) for arterial pressure monitoring. Heparin 100–200 IU/kg was administered IV before further instrumentation and was then supplemented as needed to keep an activated clotting time over 200 s. The chest was reopened through the previous sternotomy and the LIMA was dissected from its bed in a skeletonized fashion (without a muscular pedicle). An end-to-side anastomosis between the LIMA and the arterialized GCV in the anterior longitudinal sulcus was performed without the aid of cardiopulmonary bypass. The off-pump technique included insertion of an intra-venous shunt to aid in the creation of the anastomosis. This allowed oxygenated blood to reach the ischemic myocardium in a retrograde fashion. After the revascularization procedure, the animals were recovered for 8 additional weeks.

### Echocardiography

Transthoracic echocardiography was performed in all animals using an iE33 ultrasound system (Philips, Andover, MA) with an S5-I transducer, including one echocardiogram the day of the first intervention (baseline) and one echocardiogram every 2 weeks until the end of the study. All echocardiographic images were acquired with the animal placed in the supine and right lateral decubitus positions. Images were taken in the short axis and long axis views and analyzed offline by an investigator blinded to the data. EF was calculated using the Simpson's method.

### Coronary angiography

Angiograms of the LAD artery were performed in all animals every two weeks, including one at baseline and one at the end of the study. Angiograms were obtained in the postero-anterior, left anterior oblique, right anterior oblique, and lateral views using a BV Pulsera c-arm mobile x-ray system (Philips, Andover, MA).

### Blood sampling

Serial blood samples were collected via jugular puncture at baseline and then once every two weeks until the end of the study. Values of cTnI were obtained using an iSTAT handheld analyzer (Abaxis, Union City, CA).

### Heart preparation

After the animals were euthanized on the terminal day, the coronary arteries were cannulated and perfused with 0.9% sodium chloride to flush out the blood, and then with 1 L of Carson's Millonig Formalin. The hearts were kept refrigerated in the fixation solution for a minimum of 24 h before preparation for histological analysis.

### Histological preparation

Myocardial tissue samples were obtained from the area at risk of the LV free wall at approximately the same location in both animal groups for comparison. The samples were sliced under the microscope, using ordinary razor blades, into ∼3 × 3 × 3 mm uniform pieces. The samples were rinsed three times with a buffer solution, processed by dehydration in increasing concentrations of alcohol (70%, 80%, 95%, and 100%), and embedded in glycol methacrylate (JB-4 solution). The tissue blocks were placed in a specimen holder and sections of 3 µm thickness were cut using a conventional microtome (HM 340 E, Microm, Walldorf, Germany), mounted on glass slides, and stained with 0.1% Toluidine blue for assessment of myocardial and vessel damage.

### Statistical analysis

Ejection fraction (EF) and cardiac troponin I (cTnI) during six time points were analyzed using a mixed model analysis of variance (ANOVA) with Time as the within-subject variable, and Group (Group I vs. Group II) as the between subject variable. ANOVA data were analyzed using the Greenhouse-Geisser correction for violation of Mauchly's Test of Sphericity. If interaction was detected, simple main effects were analyzed using independent samples *t*-tests at each of the 6 time points. Significance was considered at *p *< 0.05.

## Results

**Figure 4:**
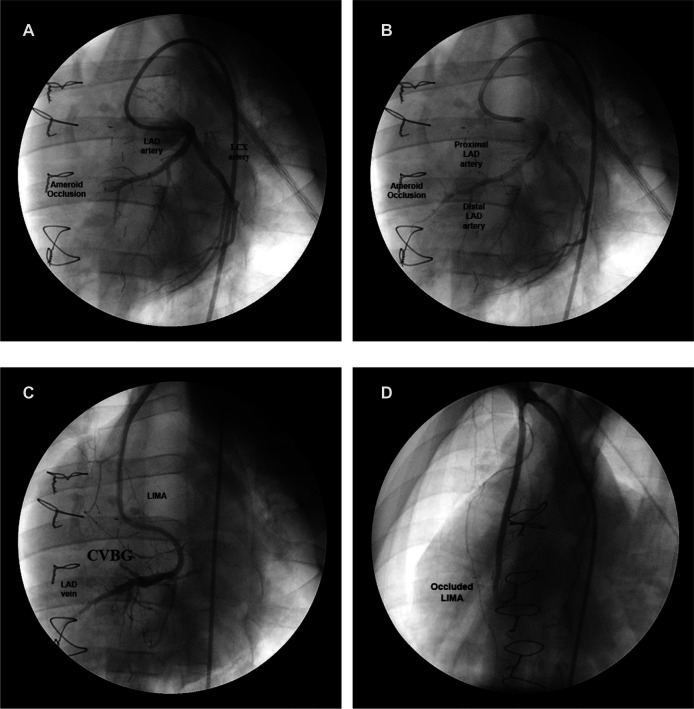
Coronary angiogram at different stages in a representative animal. (**A**) Ameroid-occluded LAD artery at early contrast injection. (**B**) Same artery a few seconds later after additional filling. (**C**) CVBG between the LIMA and the arterialized GCV two weeks after the revascularization procedure. (**D**) Occluded LIMA (graft failure) four weeks after CVBG.

All animals had total occlusion of the LAD artery, confirmed by angiography, two weeks after ameroid placement and GCV ligation/occlusion. An angiogram of the LAD artery shows the ameroid-occluded artery during early injection of contrast material (Figure 4A), and a few seconds later (Figure 4B) after additional filling. The filling with contrast of the LAD artery distal to the ameroid occlusion indicates the presence of novel “collaterals” or connections between the native arterioles and the newly arterialized venules. Figure 4C shows the CVBG between the LIMA and the GCV two weeks after the revascularization procedure. Approximately four weeks after CVBG, occluded grafts (Figure 4D) were found in all the animals in Group I possibly due to competitive flow through the arterial-newly arterialized venous “collateralization”.

**Figure 5:**
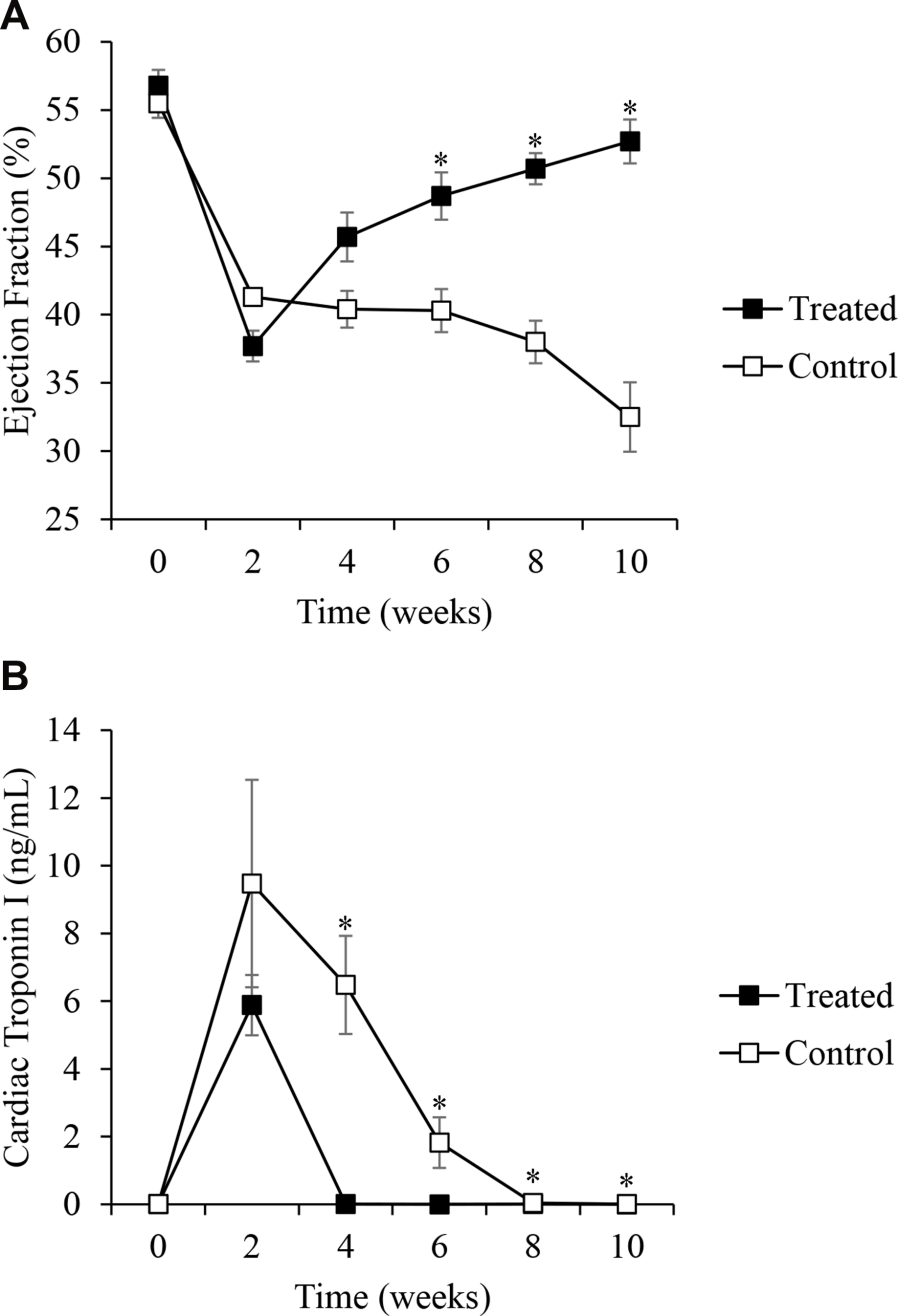
(**A**) Changes in ejection fraction from echocardiograms at different time points in the treated (*n* = 19, black squares) and control (*n* = 8, white squares) groups. *p < 0.05. Data were analyzed for statistical significance using ANOVA followed by the Greenhouse-Geisser correction method and Student's *t*-test. (**B**) Changes in cardiac troponin I levels from baseline to the end of the study in both the treated (*n* = 19, black squares) and control (*n* = 8, white squares) groups. *p < 0.05. Data were analyzed for statistical significance using ANOVA followed by the Greenhouse-Geisser correction method and Student's *t*-test.

Figure 5A shows the EF measured in both Group I and Group II. In Group I (treated group), the EF decreased from 57% ± 5% to 38% ± 5% two weeks after ameroid placement. After CVBG was performed, the EF progressively increased to 46% ± 8% (4 weeks), 49% ± 8% (6 weeks), 51% ± 5% (8 weeks), and 53% ± 7% (10 weeks). In Group II (control group), the EF dropped below normal values from 56% ± 3% at baseline to 41% ± 2% after two weeks occlusion and continued dropping to 40% ± 4% (4 weeks), 40% ± 5% (6 weeks), 38% ± 4% (8 weeks), and 33% ± 7% (10 weeks) in the surviving animals (8 out of 13). The analysis showed that there was no difference in EF at baseline and 2 and 4 weeks, but EF was significantly higher in the treated group compared to the control group at 6 weeks [(*t*_(25)_ = −2.86, *p *= 0.008), 8 weeks (*t*_(25)_ = −6.14, *p *< 0.001), and 10 weeks (*t*_(25)_ = −6.54, *p *< 0.001), Figure 5A]. The mortality (38%) in the control group, which occurred between the second- and third-weeks post-occlusion, most likely was the result of ventricular arrhythmias. The treated group had 0% mortality.

The levels of cTnI in the treated group, measured in venous blood samples, increased from 0.01 ± 0.01 ng/ml (baseline) to 5.9 ± 3.9 ng/ml two weeks after ameroid occlusion, and then reached baseline values once CVBG was performed, 0.01 ± 0.01 ng/ml (4 weeks), and 0.00 ± 0.01 ng/ml (6, 8 and 10 weeks), Figure 5B. In the control group, cTnI increased from 0.01 ± 0.01 ng/ml (baseline) to 8.29 ± 8.19 (2 weeks occlusion) and remained elevated for approximately 8 weeks (Figure 5B). The analysis showed that there was no statistically significant difference between the control and treated groups in cTnI at baseline and 2 weeks, but the treated group had significantly lower cTnI compared to the control group at 4 weeks (*t*_(24)_ = 7.63, *p *< 0.001), 6 weeks (*t*_(24)_ = 4.16, *p *< 0.001), 8 weeks (*t*_(24)_ = 8.64, *p *< 0.001), and 10 weeks (*t*_(24)_ = 2.36, *p *= .027).

**Figure 6:**
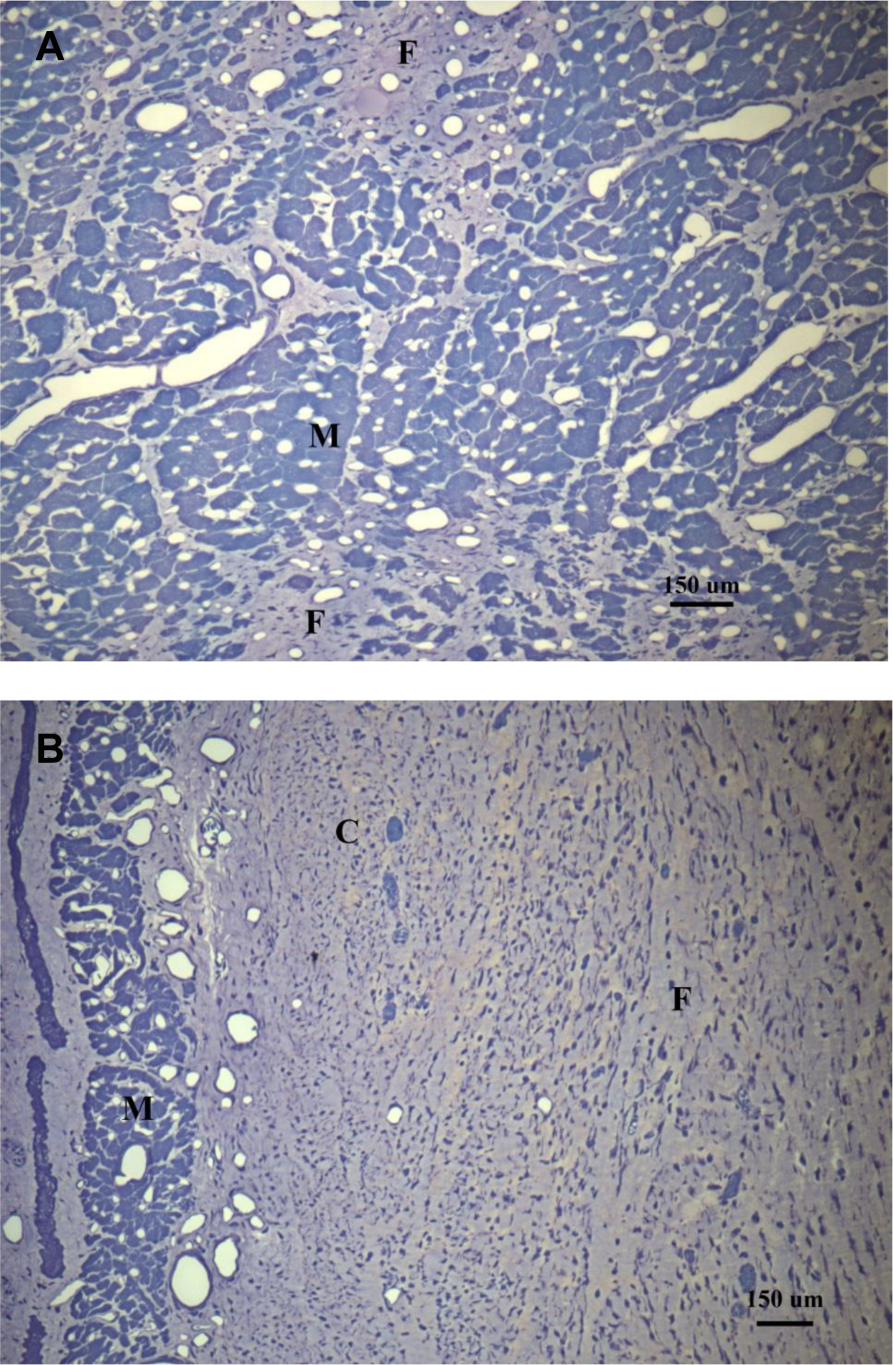
Histological images from a representative animal from comparative regions in (**A**) the treated group (toluidine blue at *X*100) showing patchy areas of fibrosis, and (**B**) the control group (toluidine blue at *X*100) where most of the myocytes have been replaced with fibrotic tissue. M, myocytes with vacuolization; F, fibrosis; C, chronic inflammation.

Macroscopic examination of the venous system in the explanted hearts did not show evidence of venous aneurysms or vein degradation. Histological examination of the myocardium in Group I (Figure 6A) demonstrated the absence of edema and hemorrhage after 8 weeks of revascularization. Only small areas of fibrosis and myocyte vacuolization were observed. In Group II (Figure 6B), there were large areas of fibrosis, necrotic tissue and chronic inflammatory infiltrate substituting the myocytes, more evident in the subendocardial region. Cross-sections of the GCV (Supplementary Material) collected two weeks after VPP device implant as preliminary data for this study, showed a completely occluded vein by thrombus formation in the proximal end of the VPP device (opposite to the CVBG), as well as wall thickness of a normal vein compared to a remoded one post VPP device implant.

Bench testing of the VPP device demonstrated similar changes to ligation of the GCV in pulse width as well as peak pressure. Percutaneous delivery of the VPP device into the GCV in the *in vivo* studies resulted in similar findings to suture ligation (Supplemental Material).

## Discussion

The major finding of this study was that selective chronic retroperfusion post 2-week arterialization of coronary veins (either through surgical ligation or percutaneous closure device) at intermediate pressures was able to recover LV myocardial function, as demonstrated by full restoration of EF in hearts with complete LAD artery occlusion. A clear decline in EF, on the contrary, was observed in the untreated animals, which persisted through the duration of the study. Furthermore, the areas at risk of infarct were substantially reduced in the treated group, and there was no edema or hemorrhage of venous vessels. In the untreated group, a mortality of 38% was observed, which has been reported in the literature (15) to be as high as 80% with LAD ameroid occlusion. In the treated group, however, the mortality was zero. The mechanism of revascularization achieved in this study was possibly the result of newly developed interconnections between the native arteries and the newly arterialized veins since intrinsic angiogenesis and collateralization are not sufficient to improve myocardial function post-ischemia (16). Local upregulation of vascular endothelial growth factor (VEGF), however, has been demonstrated in response to myocardial ischemia in swine (17). We speculate that under adequate conditions (higher pressure in the venous territory), growth factors like VEGF, promote new vessels formation in the ischemic region, between the native arterioles and the newly arterialized venules in this case.

Severe injury (edema and hemorrhage) to the coronary venous system occurs when the venous pressure exceeds 60 mmHg, as demonstrated by Hammond and associates (18). Total occlusion/ligation of the GCV did not translate into engorgement and hemorrhage because of the distinctive characteristics of the coronary venous system. The coronary venous anatomy is comprised of an elaborate network of interconnecting and redundant pathways, including inter-venous connections, Thebesian-sinus connections, and a venous plexus (19). Without these veno-venous interconnections and redundant pathways, occlusion of the GCV and CVBG would have resulted in increased pressure and flow stagnation, which was not observed. Furthermore, these adverse effects of sustained retroperfusion are also more likely to happen with global retroperfusion than with selective retroperfusion. To remedy this issue, it is necessary to gradually increase the venous pressure to allow remodeling of the venous system prior to chronic retroperfusion. We previously demonstrated (13, 14) that intermediate (∼50 mmHg) elevated venous pressure can cause functional remodeling (increase in wall thickness) of large (13) and small (14) veins without damage of the venous wall.

Selective arterialization of coronary veins at approximately 50 mmHg provides a novel approach for chronic retroperfusion without causing regional myocardial edema in the GCV territory and without affecting the venous drainage (19). Chronic retroperfusion may have significant utility to help both patients with refractory angina (despite optimal medical therapy and revascularization 20) and patients with coronary arteries not amenable to PCI or CABG (“no-option” patients 2) due to diffuse disease, recurrent re-stenotic lesions, multiple stents or failed bypass surgery.

Several related modalities of therapy like intermittent coronary sinus occlusion (ICSO), alone or in combination with retroperfusion (21) for the ischemic myocardium, has been previously investigated. A meta-analysis of seven studies evaluating the effects of ICSO demonstrated a reduction of infarct size of approximately 29%, whereas a meta-analysis of five studies analyzing the effects of ICSO and retroperfusion revealed a decrease in infarct size of approximately 39%. A comparison, however, between both groups of studies found no statistical difference (22). In the present study, we demonstrated that selective total occlusion of the GCV prior to retroperfusion allows effective revascularization of the ischemic myocardium with marked functional improvement. A small group of animals (*n* = 4) that only had LAD ameroid occlusion and GCV occlusion with VPP device (not included in this study due to the relatively small sample), showed a higher EF than the control group after the arterial occlusion. The EF, however, never returned to baseline values as in the treated group but continued to deteriorate; hence, elevation of the venous pressure is not enough to restore cardiac function.

The histological evaluation of the myocardium revealed small (patchy) areas of fibrosis and regions of myocyte vacuolization in the treated and control groups. These areas of myocyte vacuolization reflect the loss of sarcomeres due to chronic ischemia but are considered hibernating myocardium susceptible to functional recovery upon revascularization, as new sarcomeres repopulate these areas (23). Hochberg and colleagues (24) have previously demonstrated that flow through CVBG perfuses all three layers of the myocardium, but most importantly the subendocardium (Figure 6A), the most vulnerable layer to ischemia/infarction in the absence of flow (Figure 6B).

The filling with contrast, during angiography, of the LAD artery distal to the ameroid indicates the presence of “collaterals”. At the time of CVBG, the LAD artery was completely occluded and the LIMA graft was established with substantial flow. By the third to fourth week after revascularization, the LIMA was occluded. An explanation for the graft failure may be the smooth muscle cell proliferation and intimal hyperplasia at the venous edge of the anastomosis. Another explanation may be that the competitive flow due to interconnections between the native arteries and newly arterialized veins increased the LAD artery flow substantially, and in the process competitively occluded the LIMA. In other words, flow from the LIMA is no longer necessary to sustain cardiac function, which is now restored by perfusion of the ischemic myocardium via the arterial-newly arterialized vein “collateral” vessels. This phenomenon requires 3–4 weeks in swine and was very reproducible in all the treated animals.

**Figure 7:**
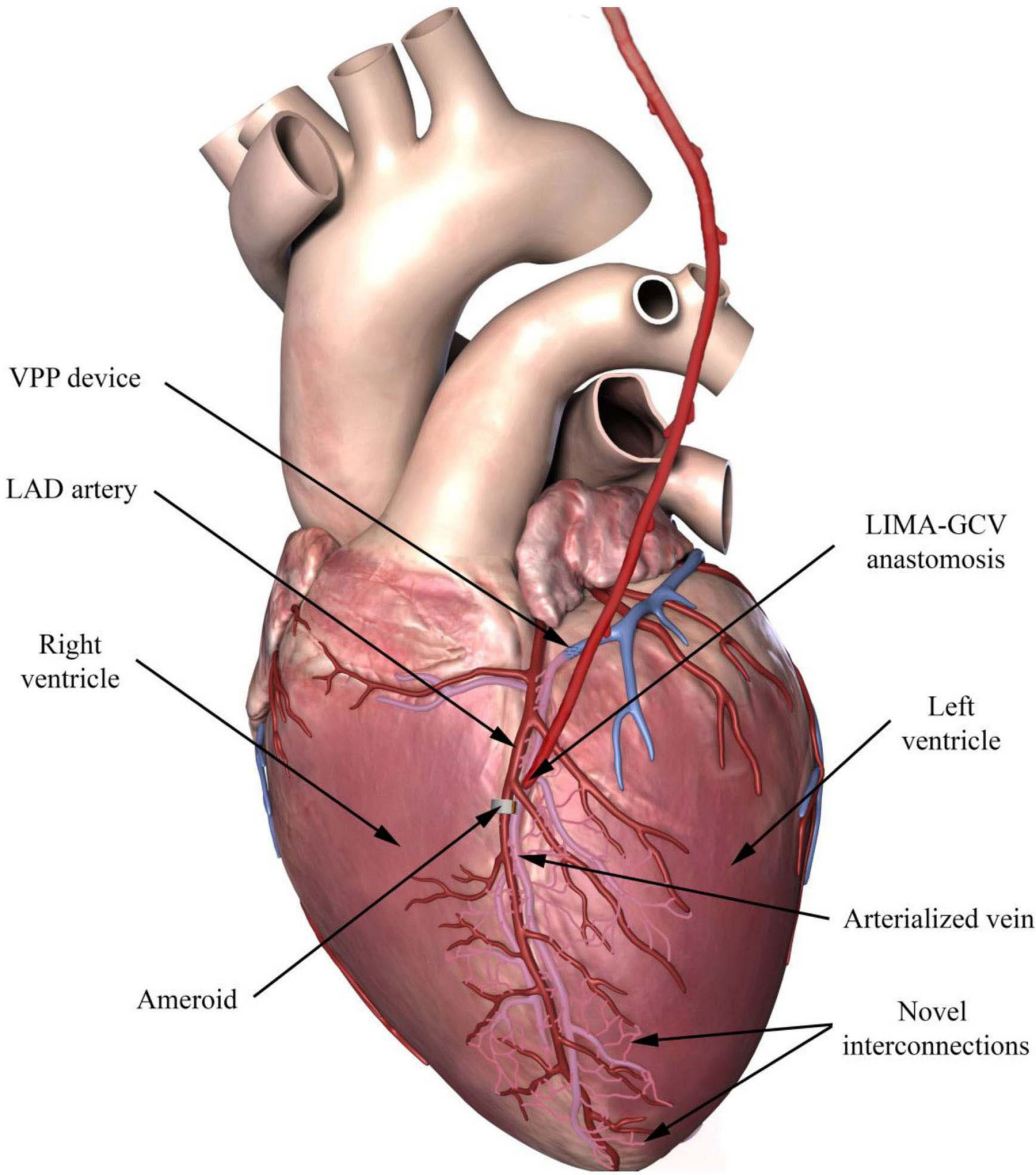
Schematic representation of the retroperfusion procedure showing the ameroid-occlusion of the LAD artery and the VPP device in the GCV, the LIMA-GCV anastomosis two weeks after and the novel interconnections (pink) between the newly arterialized veins and the native arteries.

The vasculature is an adaptable structure that is capable of biochemical, architectural, and functional adjustments in response to changes in biomechanical stimuli. Increase in pressure induced by the VPP device in the GCV leads to an increase in tensile stress, which is a stimulus for vascular growth and subsequent remodeling of the distal GCV system in a relatively short period of time. Occlusion of the GCV results in thickening of the venous wall to protect against hemorrhage during retroperfusion and stimulates local collateralization to the ischemic region. A schematic of our hypothesis of “collateralization” between the native arterioles and the newly arterialized venules is shown in Figure 7. Typically, collaterals do not form between arteries and veins as the gradients of oxygen and nutrients are typically not conducive under normal conditions. Here, we remodeled the veins, and hence, provided the gradients necessary for the development of new interconnections between these two vascular networks. These novel arteriovenous interconnections may serve to revascularize the ischemic myocardium (13).

In conclusion, chronic coronary retroperfusion via CVBG through a selectively arterialized vein in a swine model (via intermediate increase in coronary vein pressure either surgically or percutaneously) suggests that this approach may be used as an alternative form of myocardial revascularization therapy for patients in whom PCI or CABG is not feasible.

### Study limitations

The animals used in this study were free of CAD, and the ameroid occlusion model does not represent the condition of diseased coronary arteries in humans. The ameroid model, however, is the most widely used model of chronic ischemia and has the advantage of producing progressive stenosis of the artery over approximately 2 weeks (25). The gradual nature of the stenosis reduces the incidence of sudden death due to lethal arrhythmias or massive myocardial infarction when sudden acute occlusion of the artery occurs. Furthermore, this chronic model of ischemia is accepted as representative of the pathophysiologic process of human coronary stenosis. Future studies should be performed in swine with both focal and diffuse disease (26). Additionally, vein occlusion should be performed once ischemia is established to mimic the clinical scenario. We performed a simultaneous ameroid placement with vein occlusion to avoid multiple open-heart procedures in the animal.

Given that no-option patients are likely to have previous bypass grafts, a LIMA may not be feasible for CVBG, and in such cases, a saphenous vein graft (SVG) would be an alternative. Future animal studies with SVG CVBG will be implemented to evaluate the efficacy under a variety of clinically relevant conditions.

In the present study we did not include a cohort with CVBG to a non-arterialized vein given that previous studies in humans (27) and dogs (28) have demonstrated that subjecting the normal venous system to arterial pressure leads to myocardial edema and hemorrhage in the long term with high mortality due to congestion of coronary circulation and heart. We assumed these historic controls for our hypothesis given that the outcome of CVBG on arterialized veins was very positive in the present study. Given the large number of animals (*n* = 32) used in the current study, we opted to conserve resources by using historic controls.

Future studies on percutaneous delivery of the VPP device in larger number of animals are needed for device optimization prior to first in human. Our bench studies have demonstrated that the device increases pulse width and peak pressure, both of which are key to successful redistribution of endocardial blood flow and arterialization of venous vessels. The *in vivo* studies have shown similar end results between surgical suture ligation and percutaneous VPP device delivery, with the latter being the more desirable approach to vein remodeling prior to venous retroperfusion.

The improvement in LV function demonstrated by the increase in EF, the decrease in the concentrations of cTnI, and the histological findings are all suggestive of restoration of myocardial oxygen supply. Clearly, this evidence demonstrates that chronic selective retroperfusion of arterialized veins prevents deterioration of the myocardium. Furthermore, the lack of mortality in this group also supports this conclusion. Future myocardial perfusion should be assessed by SPECT or PET under baseline and stress conditions to assess the ability of the revascularization to provide flow reserve. MRI studies in this model could also provide insights into the infarct regions and myocardial function.

The presence of channels between the native arteries and the newly arterialized veins needs to be identified. Future immunostaining studies, including the expression of ephrinB2 in arteries and EphB4 in veins, are required to demonstrate the mechanisms of this novel form of “collateralization”.

## Data Availability

The raw data supporting the conclusions of this article will be made available by the authors, without undue reservation.

## Supplementary material

Supplementary Figure S1 Bench testing of VPP device. Bench studies were performed to evaluate the ability of the VPP device to achieve a similar degree of elevations in pressure and pulsatility obtained by vein ligation13,14. The device was collapsed into a delivery system and placed in a pulse duplicator (BDC Labs, Denver, CO) programmed to generate venous flow and pressure profiles. A) Changes in pulse width using a pulse duplicator. *n* = 3/group. *p* < 0.05. Data were analyzed for statistical significance using Student's *t*-test. B) Representative tracing of maximum pressure with VPP device vs. control.

Supplementary Figure S2 A) GCV specimen showing complete occlusion 14 days post VPP device implant. B) Histologic image of the GCV completely occluded by thrombus formation, H&E staining at X40. C) GCV from a control animal showing a thin wall vessel, Toluidine blue at X60. D) GCV post VPP device implant, showing significant smooth muscle hypertrophy of the medial layer, α-actin staining at X100.
