# Supplementary figures and images for: Pre-arterialization of coronary veins prior to retroperfusion of ischemic myocardium: percutaneous closure device
Source: Front Cardiovasc Med. 2023 Sep 18;10:1208903. doi: 10.3389/fcvm.2023.1208903 (PMC10543752; doi:10.3389/fcvm.2023.1208903)

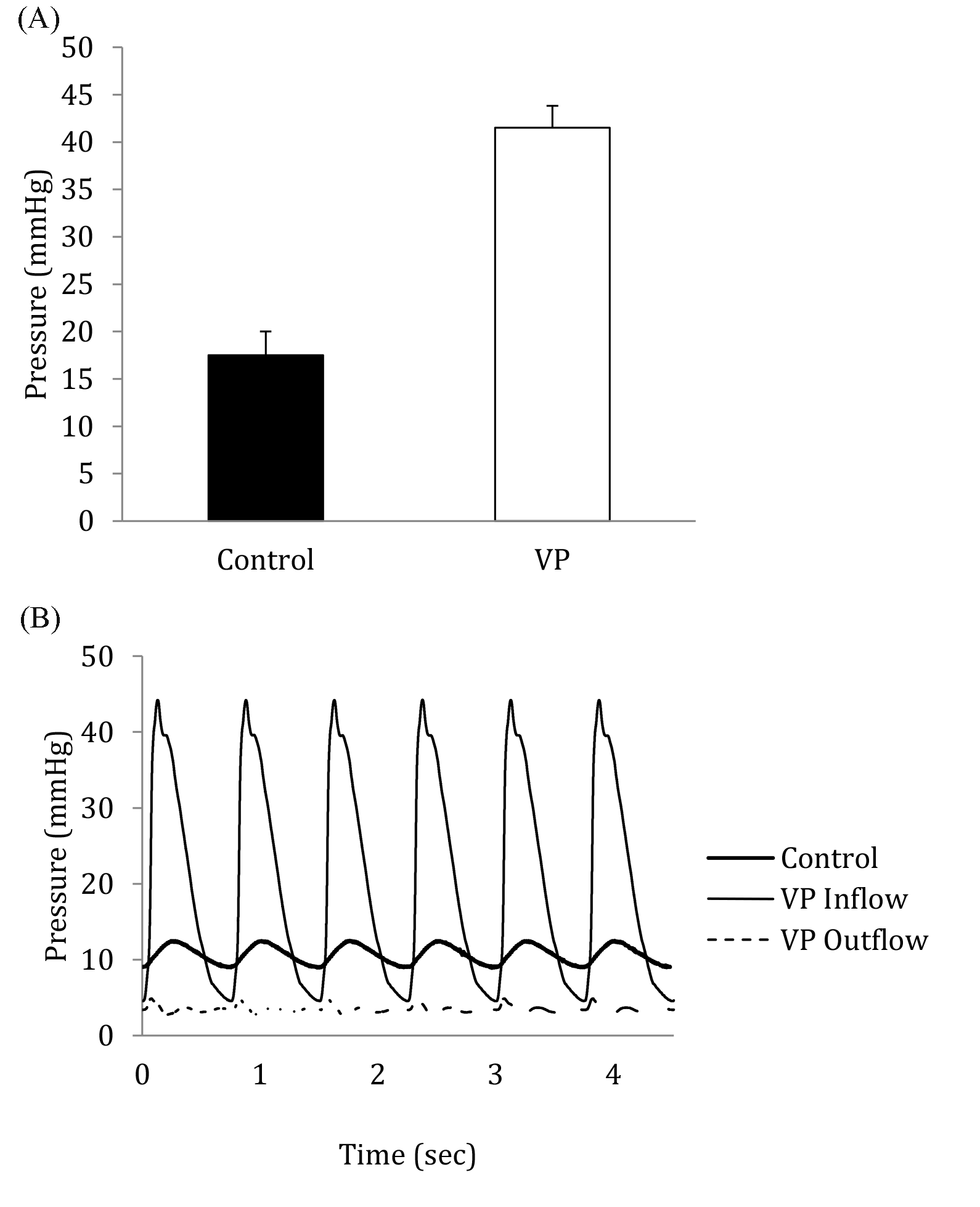

Supplement: Supplementary Figure S1 — Bench testing of VPP device. Bench studies were performed to evaluate the ability of the VPP device to achieve a similar degree of elevations in pressure and pulsatility obtained by vein ligation13,14. The device was collapsed into a delivery system and placed in a pulse duplicator (BDC Labs, Denver, CO) programmed to generate venous flow and pressure profiles. A) Changes in pulse width using a pulse duplicator. n = 3/group. p < 0.05. Data were analyzed for statistical significance using Student's t-test. B) Representative tracing of maximum pressure with VPP device vs. control. [file Image1.tif]

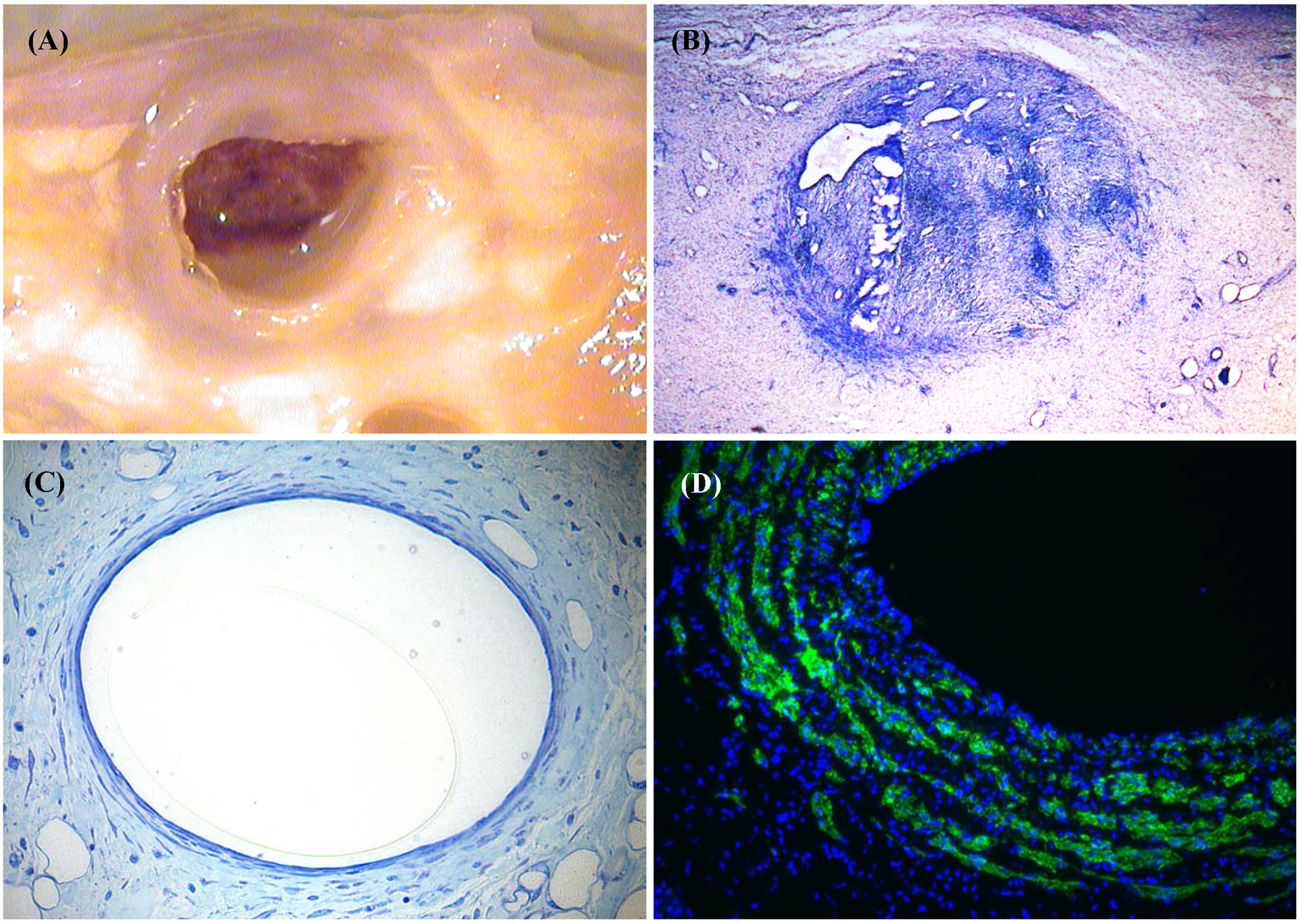

Supplement: Supplementary Figure S2 — A) GCV specimen showing complete occlusion 14 days post VPP device implant. B) Histologic image of the GCV completely occluded by thrombus formation, H&E staining at X40. C) GCV from a control animal showing a thin wall vessel, Toluidine blue at X60. D) GCV post VPP device implant, showing significant smooth muscle hypertrophy of the medial layer, α-actin staining at X100. [file Image2.tif]
